# Influence of *Bxpel1* Gene Silencing by dsRNA Interference on the Development and Pathogenicity of the Pine Wood Nematode, *Bursaphelenchus xylophilus*

**DOI:** 10.3390/ijms17010125

**Published:** 2016-01-19

**Authors:** Xiu-Wen Qiu, Xiao-Qin Wu, Lin Huang, Jian-Ren Ye

**Affiliations:** 1 Collaborative Innovation Center of Sustainable Forestry in Southern China, College of Forestry, Nanjing Forestry University, Jiangsu Key Laboratory for Prevention and Management of Invasive Species, Nanjing 210037, China; qiuxiuwen3@163.com (X.-W.Q.); xqwu@njfu.edu.cn (X.-Q.W.); Lhuang@njfu.edu.cn (L.H.); 2 Poyang Lake Eco-economy Research Center, Jiujiang University, Jiujiang 332005, China

**Keywords:** *Bursaphelenchus xylophilus*, pathogenicity, pectate lyase, RNA interference (RNAi)

## Abstract

As the causal agent of pine wilt disease (PWD), the pine wood nematode (PWN), *Bursaphelenchus xylophilus*, causes huge economic losses by devastating pine forests worldwide. The pectate lyase gene is essential for successful invasion of their host plants by plant-parasitic nematodes. To demonstrate the role of pectate lyase gene in the PWD process, RNA interference (RNAi) is used to analyze the function of the pectate lyase 1 gene in *B. xylophilus* (*Bxpel1*). The efficiency of RNAi was detected by real-time PCR. The result demonstrated that the quantity of *B. xylophilus* propagated with control solution treatment was 62 times greater than that soaking in double-stranded RNA (dsRNA) after *B. xylophilus* inoculation in *Botrytis* *cinerea* for the first generation (F1). The number of *B. xylophilus* soaking in control solution was doubled compared to that soaking in *Bxpel1* dsRNA four days after inoculation in *Pinus* *thunbergii.* The quantity of *B. xylophilus* was reduced significantly (*p* < 0.001) after treatment with dsRNAi compared with that using a control solution treatment. *Bxpel1* dsRNAi reduced the migration speed and reproduction of *B. xylophilus* in pine trees. The pathogenicity to *P. thunbergii* seedling of *B. xylophilus* was weaker after soaking in dsRNA solution compared with that after soaking in the control solution. Our results suggest that *Bxpel1* gene is a significant pathogenic factor in the PWD process and this basic information may facilitate a better understanding of the molecular mechanism of PWD.

## 1. Introduction

Pine wilt disease (PWD), which is caused by the pine wood nematode (PWN), *Bursaphelenchus xylophilus* (Steiner & Buhrer) Nickle, is one of the most serious diseases that damages coniferous forests [1,2,3,4]. PWD causes huge economic losses and has severe environmental and social impacts in Asia, causing the death of millions of pine trees [5]. The PWN is carried from one tree to another by the longhorn beetle, *Monochamus alternatus* [6,7,8]. The nematode feeds mainly on the xylem ray parenchyma cells of healthy pine trees [9]. At present, there are various control methods to prevent the dispersal of PWD and many studies have focused on the pathogenic mechanism of *B. xylophilus* by sequencing analysis [10,11,12].

When invading their hosts, PWNs need to break down the cell wall barrier in pine trees. Pectin is an essential component of the plant cell wall, thus PWNs need to secrete a mixture of cell wall-degrading enzymes to macerate the complex structure of pectin and other components of the cell wall [13,14,15]. Pectate lyases are considered to be significant pathogenic factors in phytopathogens and are essential for plant-parasitic nematodes to successfully invade their host [16,17].

RNA interference (RNAi) is an effective method for investigating the functions of genes because it degrades a specific mRNA sequence via homologous double-stranded RNA interference (dsRNAi) to silence a gene’s function at the post-transcriptional level [18,19,20]. RNAi has been used to study the functions of genes in a number of nematodes, such as root-knot nematodes [21,22,23] and *B. xylophilus* [24]. Niu *et al.* [25] investigated the effect of *Mi-Rpn7* silencing on the motility of the root-knot nematode *Meloidogyne incognita* by RNAi and found that *Mi-Rpn7* gene silencing in *M. incognita* second-stage juvenile nematodes reduced its infectivity and motility. Cheng *et al.* [26] demonstrated that the cellulase gene of *B. xylophilus* plays a critical role in degrading cellulose in the plant cell wall, thereby influencing feeding, development, and propagation via dsRNAi. However, little is known about the functions of pectate lyase genes in *B. xylophilus* during the PWD process.

The PWN penetrates plant tissues and feeds on cytoplasm from the plant cell, which subsequently causes the death of host cells and ultimately leads to the wilting of pine trees [27]. The plant cell wall is a powerful barrier that resists the penetration of nematodes [26,28]; therefore, it is necessary to secrete plenty of pectate lyases to facilitate feeding and movement in pine trees. Kikuchi *et al.* [29] cloned a pectate lyase gene (*Bxpel1*) from the PWN and their results showed that *B. xylophilus* secreted pectate lyases into plant tissues to facilitate its feeding and migration in pine trees. However, the relationship between the pectate lyase gene and the pathogenicity of *B. xylophilus* is still unclear*.* The *Bxpel1* gene was selected because it is highly expressed during the PWD process [27]. In the present study, we successfully silenced the *Bxpel1* gene by RNAi. Thus, the goal of this study was to analyze the effects of silencing the pectate lyase gene by RNAi on the propagation, migration, and pathogenicity of *B. xylophilus*.

## 2. Results

### 2.1. Morphology of B. xylophilus after Double-Stranded RNA Interference (dsRNAi)

**Figure 1:**
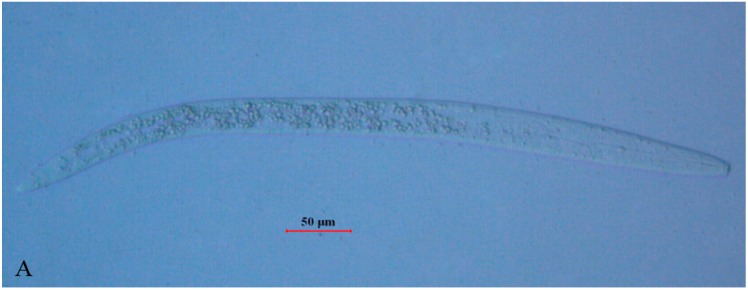

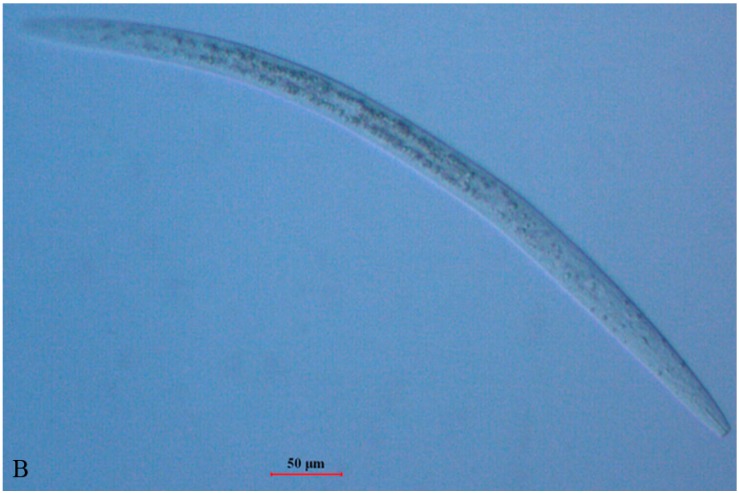
The phenotype of *B. xylophilus* after soaking in doble distilled water (ddH_2_O) (**A**) and control solution (**B**) for 48 h. Scale bars = 50 μm.

**Figure 2:**
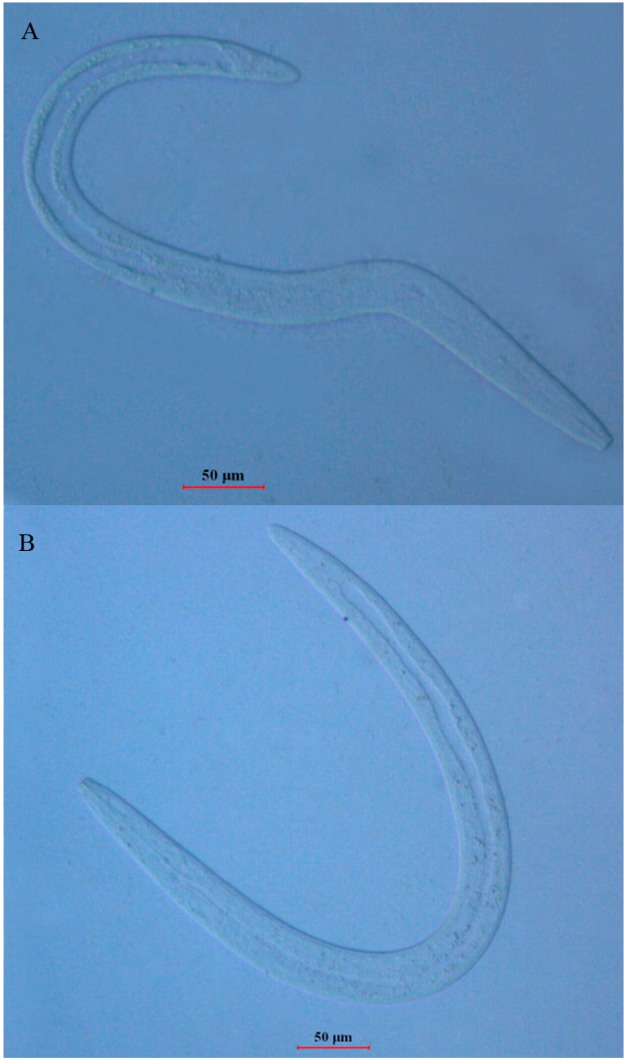
The phenotype of *B. xylophilus* after soaking in *Bxpel1* double-stranded RNA (dsRNA) for 48 h (**A**,**B**). Scale bars = 50 μm.

We performed an experiment to determine the differences in morphology of *B. xylophilus* with and without dsRNAi in order to elucidate the effects of RNAi on *B. xylophilus*. Figure 1A,B and Figure 2 show the morphology of *B. xylophilus* soaking in the doble distilled water (ddH_2_O), control solution and *Bxpel1* double-stranded RNA (dsRNA) for 48 h, respectively. Some lipid droplets were found on the gut wall of *B. xylophilus* in the ddH_2_O and control solution treatments, but not in *Bxpel1* dsRNA treatment. Results indicated that *Bxpel1* dsRNA had a significant effect on the morphology of *B. xylophilus*.

### 2.2. Propagation of B. xylophilus on Fungal Culture after dsRNAi

**Figure 3:**
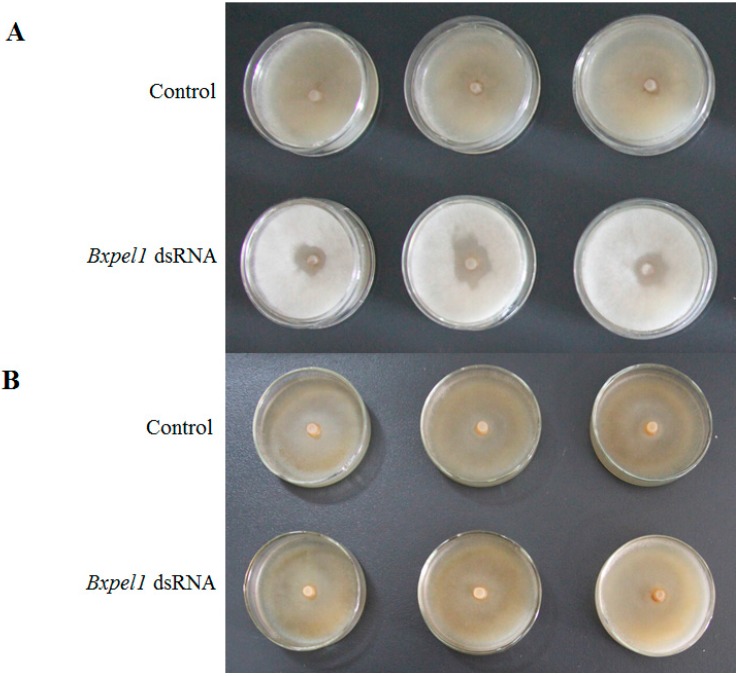
Effects of double-stranded RNA interference (dsRNAi) on *B. xylophilus* propagation. (**A**) The growth of the first generation of *B. xylophilus* on *B. cinerea*; (**B**) The growth of the second generation of *B. xylophilus* on *B. cinerea.*

**Figure 4:**
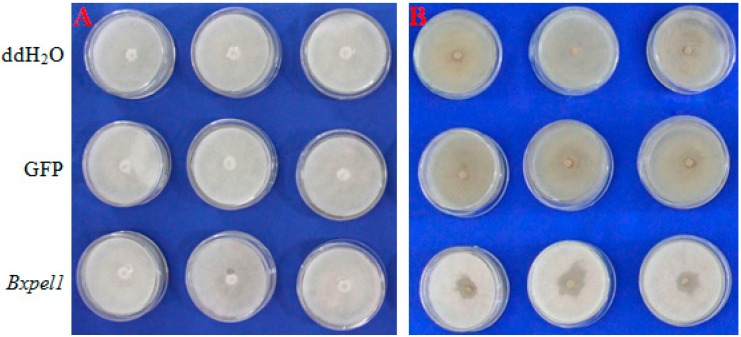
Effects of *GFP* gene dsRNA on propagation of *B. xylophilus*. (**A**) *B. xylophilus* inoculating on *B. cinerea* on the first day; (**B**) *B. xylophilus* inoculating on *B. cinerea* on the seventh day*.*

We used potato dextrose agar (PDA) plates inoculated with *Botrytis cinerea* to test the effects of dsRNAi on the propagation of *B. xylophilus* at five days after inoculation (Figure 3); the results showed that *Bxpel1* RNAi strongly affected the first generation (F1). The nematodes soaked in the control solution grew more rapidly than those soaked in *Bxpel1* dsRNA solution at five days after inoculation on *B. cinerea*. However, *Bxpel1* dsRNAi had no significant effect on the second generation (F2) when the F1 was inoculated onto new *B. cinerea* after five days. A similar phenomenon was also found from F3 to F7 generations. To test the effect of non-endogenous genes on nematodes, *B. xylophilus* was soaked in green fluorescent protein (*GFP*) gene dsRNA. Results showed that there was no effect of *GFP* gene dsRNA on the propagation of *B. xylophilus* on *B. cinerea* (Figure 4).

**Figure 5:**
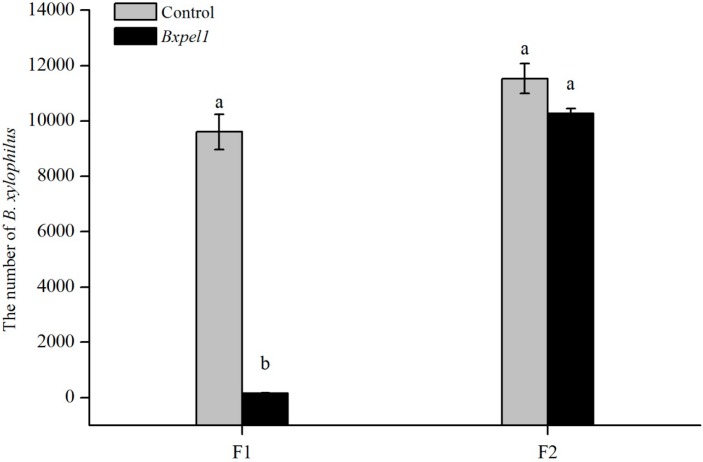
Effects of dsRNAi on *B. xylophilus* reproduction. Vertical bars indicate standard deviation of the means (*n* = 3), and different letters indicate significant differences at a level of *p* < 0.05 based on Tukey’s test. F1: first generation; F2: second generation.

There was a significant difference between the number of nematodes in the control solution and that in the dsRNAi solution in the F1. The number of F1 nematodes was about 9600 with the control solution treatment; whereas, the number was 155 after *Bxpel1* dsRNA treatment (Figure 5). However, there was no significant difference between the numbers after treatment with the control solution and dsRNAi solution in the F2 generation, *i.e.*, the average number of nematodes with the control solution treatment was 11,533 and that with the *Bxpel1* dsRNA treatment was 10,266 (Figure 5).

In general, it was found that *Bxpel1* dsRNAi had significant effects on the propagation of the F1 of *B. xylophilus*. The quantity of *B. xylophilus* propagated with the control solution treatment was 62 times greater than that in the *Bxpel1* dsRNA treatment. The effect of *Bxpel1* dsRNAi occurred during the early stages of interference (at about five days) and disappeared in the late stages.

### 2.3. Efficiency of RNAi in B. xylophilus

**Figure 6:**
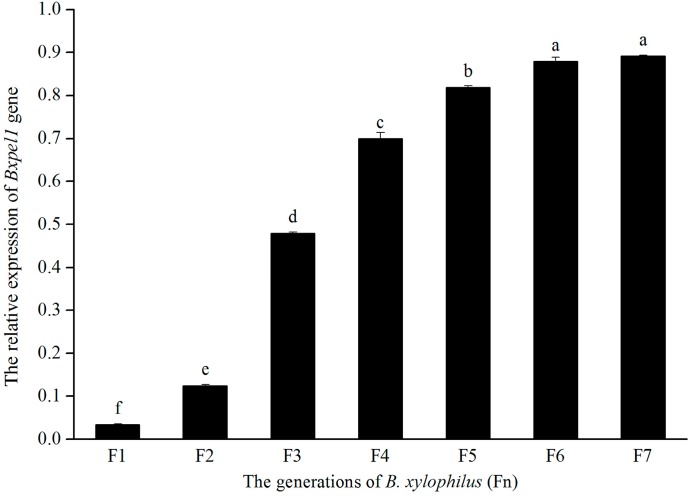
Expression of the *Bxpel1* gene in *B. xylophilus.* Vertical bars indicate standard deviation of the means (*n* = 3), and different letters indicate significant differences at a level of *p* < 0.05 based on Tukey’s test. F3: third generation; F4: fourth generation; F5: fifth generation; F6: sixth generation; F7: seventh generation.

RT-PCR was used to detect the effects of dsRNAi on *Bxpel1* in *B. xylophilus*. Figure 6 shows the expression of the *Bxpel1* gene in *B. xylophilus* from F1 to F7 generations after dsRNAi. The actin gene was used as a control. The results indicate that the expression levels of the *Bxpel1* gene were 0.034, 0.125, 0.479, 0.699, 0.819, 0.879, and 0.891 in each generation compared with the actin gene. Thus, *Bxpel1* dsRNAi had a significant inhibitory effect on F1 and F2 in *B. xylophilus*, but the effect was minimal after F3. Therefore, the expression of *Bxpel1* recovered in *B. xylophilus* and the effect of dsRNAi disappeared after F5. The RT-PCR results showed that double-stranded RNAi (dsRNAi) had a suppressive effect on the expression of *Bxpel1* gene on the F1 and F2 of *B. xylophilus*, but not for the later generations.

### 2.4. Mortality of Pinus thunbergii after Inoculating B. xylophilus with dsRNAi

**Figure 7:**
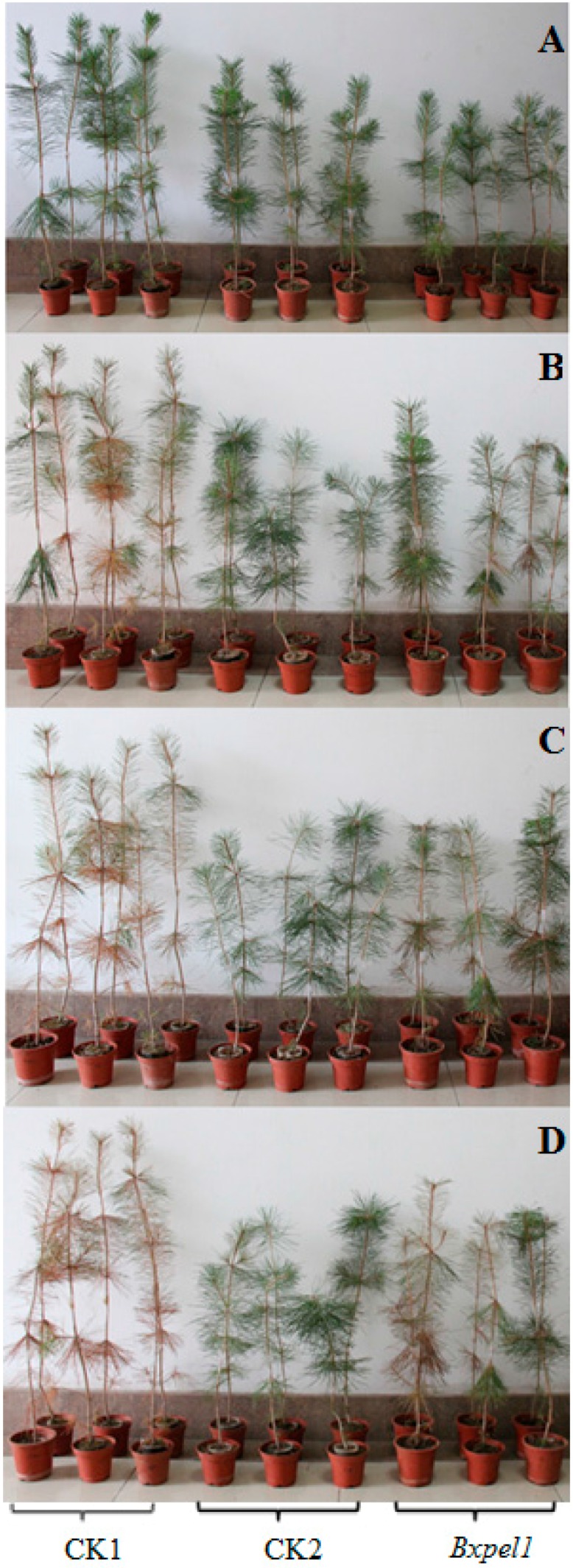
Wilting symptoms development of *P. thunbergii* after inoculation with *B. xylophilus* at 1 day (**A**); 10 days (**B**); 20 days (**C**); and 30 days (**D**). The pine trees were inoculated with PWNs that had been soaked in control solution (CK1), ddH_2_O (CK2), or *Bxpel1* dsRNA (*Bxpel1*).

In *B. xylophilus*, the *Bxpel1* gene plays an important role in lethal time for the host. Some *P*. *thunbergii* plants were found wilted within 10 days after inoculation with *B. xylophilus* soaked in control solution and some of the *P*. *thunbergii* needles turned yellow, whereas the needles of *P*. *thunbergii* remained green after inoculation with ddH_2_O and *Bxpel1* dsRNA (Figure 7B). Almost all the pine trees wilted within 20 days after inoculation with *B. xylophilus* soaked in the control solution and the mortality rate of *P*. *thunbergii* was almost 90% (Figure 7C). By contrast, only a few *P*. *thunbergii* became yellow at 20 days after inoculation with *Bxpel1* dsRNA-treated *B. xylophilus* (Figure 7C). All the pine trees died after 30 days following treatment with the control solution, whereas the *P*. *thunbergii* mortality rate was almost 50% after *Bxpel1* dsRNA treatment (Figure 7D). *P*. *thunbergii* grew well throughout the experiment process when inoculated with ddH_2_O.

### 2.5. Migration and Reproduction of B. xylophilus in P. thunbergii after Soaking in dsRNA

We determined the number of *B. xylophilus* at different sampling times to elucidate the migration of *B. xylophilus* in pine trees after soaking in *Bxpel1* dsRNA. Table 1 shows the numbers of *B. xylophilus* in pine trees following treatment with the sterile water and *Bxpel1* dsRNA after inoculation. For sterile water treatment, the number of *B. xylophilus* was 66 at 5 cm above the site of inoculation and 300 at 5 cm below the site at day four after inoculation. The number of *B. xylophilus*-treated sterile water was much lower at 10–15 cm than that at 5 cm below the inoculation site and there were no nematodes in other parts of the pine trees. PWNs were detected in all parts of the pine trees at eight days after inoculation and the number of *B. xylophilus* reached the maximum at day 20. For *Bxpel1* dsRNA treatment, the migration of *B. xylophilus* in pine trees was similar to that when soaked in the sterile water at day four after inoculation. However, the number of *B. xylophilus* in pine trees soaking in *Bxpel1* dsRNA was significantly (*p* < 0.001; Tukey’s test) lower than that in the sterile water at day eight after inoculation. PWNs were detected in all parts of *P. thunbergii* at day 12 after inoculation when soaked in *Bxpel1* dsRNA.

Our results showed that the PWNs migrated in two directions (both up and down) after being inoculated into pine trees, where the speed of downward migration was faster than that of upward migration. *Bxpel1* dsRNA treatment affected the speed of migration and multiplication of *B. xylophilus* in *P. thunbergii*. The total number of *B. xylophilus* following soaking in sterile water was 415 at four days after inoculation, whereas the total number after soaking in *Bxpel1* dsRNA was 150. The total number of *B. xylophilus* after treatment with the sterile water was 1520 at eight days after inoculation, whereas the total number following *Bxpel1* dsRNA treatment was 431. The reproduction quantity of *B. xylophilus* with *Bxpel1* dsRNA treatment reached 6908 at 12 days after inoculation. Our results showed that the migration speed in pine trees of *B. xylophilus* following soaking in *Bxpel1* dsRNA was slower than that of *B. xylophilus* in the sterile water. The amount of *B. xylophilus* that propagated in pine trees was lower following soaking in *Bxpel1* dsRNA compared with that after soaking *B. xylophilus* in the sterile water.

**Table 1:** Migration and reproduction of *B. xylophilus* after inoculation into *P. thunbergii*.

Distance	Treatment	4 Days	8 Days	12 Days	16 Days	20 Days	24 Days
Up-5 cm	Control	66 ± 11 a ^A^	222 ± 27 a	3022 ± 279 a	6133 ± 321 a	14,933 ± 789 a	13,562 ± 280 a
	*Bxpel1*	39 ± 7 b	122 ± 9 b	1288 ± 85 b	2933 ± 152 b	3511 ± 189 b	5362 ± 157 b
Up-10 cm	Control	0	177 ± 7 a	1466 ± 32 a	4333 ± 306 a	15,000 ± 392 a	15,244 ± 398 a
	*Bxpel1*	0	44 ± 4 b	822 ± 55 b	2577 ± 103 b	6422 ± 270 b	8412 ± 176 b
Up-15 cm	Control	0	133 ± 27	1977 ± 24 a	2244 ± 205 b	6866 ± 376 b	10,228 ± 1461 a
	*Bxpel1*	0	0	466 ± 26 b	3000 ± 160 a	7700 ± 102 a	9026 ± 248 a
Up-20 cm	Control	0	67 ± 13	3977 ± 207 a	4866 ± 36 a	2311 ± 185 b	1094 ± 123 b
	*Bxpel1*	0	0	200 ± 19 b	2311 ± 139 b	5022 ± 220 a	4866 ± 108 a
Down-5 cm	Control	300 ± 19 a	424 ± 22 a	2844 ± 97 a	6177 ± 213 a	16,000 ± 475 a	12,123 ± 522 a
	*Bxpel1*	83 ± 14 b	177 ± 18 b	1355 ± 66 b	3088 ± 125 b	6822 ± 393 b	10,482 ± 545 b
Down-10 cm	Control	38 ± 9 a	211 ± 14 a	3777 ± 205 a	8511 ± 506 a	8866 ± 397 a	10,486 ± 1443 a
	*Bxpel1*	28 ± 3 a	55 ± 9 b	711 ± 23 b	3488 ± 25 b	8855 ± 242 a	10,424 ± 587 a
Down-15 cm	Control	11 ± 3	188 ± 43 a	3022 ± 82 a	15,488 ± 319 a	16,400 ± 265 a	15,724 ± 860 a
	*Bxpel1*	0	33 ± 2 b	777 ± 42 b	3244 ± 93 b	7848 ± 454 b	12,111 ± 246 b
Down-20 cm	Control	0	98 ± 8	5177 ± 152 a	12,177 ± 1223 a	10,600 ± 1651 a	11,806 ± 765 a
	*Bxpel1*	0	0	2066 ± 284 b	3478 ± 139 b	4244 ± 304 b	8711 ± 126 b

Control represents *B. xylophilus* soaked in sterile water, *Bxpel1* represents *B. xylophilus* soaked in *Bxpel1* dsRNA solution. The numbers in the first column represent the distance from the inoculation sites, the other numbers in the table represent the amount of *B. xylophilus* within different treatments, and the letters indicate the significant differences between control and *Bxpel1* treatments at *p* < 0.05 based on independent-samples *t*-test. ^A^ Means ±standard deviation (*n* = 3).

## 3. Discussion

RNAi is a cellular mechanism that allows genes to be silenced by dsRNA molecules [30]. RNAi was first discovered in *Caenorhabditis elegans* by Guo and Kemphues in 1995 [31]. RNAi has been reported to reduce the expression of genes and to be a powerful tool for studying gene functions in nematodes [30]. The successful application of RNAi has been confirmed in many plant parasitic nematodes, including *Meloidogyne incognita* [32], *Radopholus similis* [33], and *B. xylophilus* [26,34,35]. The molecular mechanism of PWD is still unclear. Thus, RNAi can be used to study the functions of genes in *B. xylophilus* and to investigate the interactions between *B. xylophilus* and pine trees [36].

Our results showed that *GFP* gene dsRNA and control solution had no effect on the morphology of *B. xylophilus*. However, lipid droplets of *B. xylophilus* disappeared in the *Bxpel1* dsRNA treatment. A recent study demonstrated that soaking in a cellulase gene dsRNA solution reduced the reproduction of *B. xylophilus* on PDA plates inoculated with *B. cinerea* [24]. Wang *et al.* [37] also found that silencing of the arginine kinase gene reduced reproduction by *B. xylophilus*. In the present study, the effect of RNAi silencing of the *Bxpel1* gene was tested on reproduction by *B. xylophilus.* Our results showed that the propagation of the F1 was significantly reduced (Figure 5), which is consistent with the results of Ma *et al.* [24]. The explanation for this may be that knockdown of the *Bxpel1* gene significantly influenced the development of *B. xylophilus*, ultimately affecting the reproduction of nematodes. The significant suppression of the expression of the *Bxpel1* in the F1 (Figure 6) and the changes in morphology probably resulted in the reduced propagation by *B. xylophilus*. Interestingly, we inoculated *B. xylophilus* soaked in *Bxpel1* gene dsRNA solution from the F2 to the F7 on *B. cinerea*, and we found that the propagation of *B. xylophilus* tended to recover after the F2 (Figure 5). Our RT-PCR results also demonstrated that the expression of the *Bxpel1* gene tended to recover from the F2 to the F7. Therefore, the *Bxpel1* gene is a key factor that regulates the propagation of the first generation but not in the later generations of *B. xylophilus*.

Park *et al.* [34] reported that the average mRNA expression levels of four genes (*Bx-myo-3*, *Bx-tmy-1*, *Bx-hsp-1*, *Bx-cyc-2.1*) were reduced by 35% (±7%) after RNAi. Cheng *et al.* [26] examined the efficiency of dsRNA interference against the cellulase gene by soaking, and demonstrated that dsRNA could be delivered effectively into *B. xylophilus* to induce post-transcriptional targeted-gene silencing in the soaked nematodes. However, the efficiency of dsRNA interference by soaking was not clear in the offspring. In the present study, the mRNA expression levels of the *Bxpel1* gene in F1, F2, and F3 generations decreased by approximately 95%, 84%, and 51%, respectively (Figure 6), but the mRNA expression level was >70% after the F4 generation. These results indicate that the mRNA expression level decreased significantly in the F1 and F2 generations but not in later generations. These results are consistent with those reported by Grishok *et al.* [38], who showed that the activities of genes subjected to interference with dsRNA recovered in the F2 and later generations.

We found that the *Bxpel1* gene expression level decreased significantly in the F1 and F2 generations due to RNAi but it gradually recovered later (Figure 6). The PWNs soaked in dsRNA required longer time to migrate throughout entire pine trees (Table 1). The reproduction of nematodes subjected to RNAi declined in pine trees compared with those in the control solution (Table 1). These results are consistent with those obtained by Bakhetia *et al.* [39], who showed that the RNAi silencing of cell wall degradation-related genes reduced the ability to locate and invade the host.

Kikuchi *et al.* [29] cloned and characterized pectate lyase genes in PWN, where they found that the pectate lyases could be secreted into plant tissues to facilitate feeding and migration in pine trees by *B. xylophilus*. Their results indicated that pectate lyases are distributed widely in *B. xylophilus* and that they have significant effects on plant–nematode interactions. The plant cell wall plays an important role in resisting invasion of *B. xylophilus*. Thus, PWNs need to produce a series of cell wall-degrading enzymes to break down the barrier and successfully invade pine trees [40,41,42]. In our previous study, the expression of pectate lyase genes was found to be upregulated six times compared to that of the actin gene after *B. xylophilus* invaded pine trees [27], indicating that the *Bxpel1* gene plays a critical role in the pathogenic process for *B. xylophilus*. In the present study, RNAi was used to investigate the effects of the *Bxpel1* gene on the pathogenicity of PWN. Our results showed that the pathogenicity of *B. xylophilus* decreased by 40% when the *Bxpel1* gene was silenced by RNAi. Therefore, it is speculated that the slower migration speed and reduced pathogenicity of *B. xylophilus* treated with pectate lyase dsRNA were due to the reduced reproduction in *P. thunbergii*. Our study provides insights into the function of the *Bxpel1* gene during the PWD process. However, the underlying mechanistic details of PWD still remain unclear, particularly the roles of pathogenic genes in *B. xylophilus*. Thus, further studies are needed to investigate and verify the functions of the *Bxpel1* gene by using other molecular methods.

## 4. Experimental Section

### 4.1. Biological Materials

The *B. xylophilus* isolate AMA3cl was obtained using Baermann funnels from *P. thunbergii* samples collected in Maanshan City, Anhui Province, China. *B. xylophilus* was cultured on PDA plates covered with a colony of *B. cinerea* at 25 °C for 5 days and isolated with Baermann funnels. The nematodes were then collected by centrifugation at 3000 rpm for 5 min. We isolated a large amount of the second stage juveniles by 0.3% carboxymethyl cellulose sodium salt (CMC) solution [43].

*P. thunbergii* seedlings (3 years old) were obtained from the nursery at the forest protection station of Nanjing Forest University and transplanted into pots (25 cm in diameter, 20 cm in height), which were watered every other day. The heights of the seedlings were 70–110 cm. The seedlings were grown in an air-conditioned greenhouse with a relative humidity of 70% and a photoperiod of 14 h day (25 °C) and 10 h night (20 °C).

### 4.2. Total RNA Preparation and cDNA Synthesis

The total RNA was extracted from nematodes using an RNAprep Kit (Tiangen, Beijing, China) and purified with an RNAclean Kit (Tiangen, Beijing, China). Total RNA was detected using a spectrophotometer and its quality was examined by electrophoresis on a 1.5% agarose gel. The cDNA was synthesized using a Prime Script 1st strand cDNA synthesis Kit (TaKaRa, Shuzo, Japan) according to the manufacturer’s instructions.

### 4.3. dsRNA Synthesis and in Vitro RNAi

The synthesized cDNA was used as the template for dsRNA synthesis. A fragment (790 bp) of pectate lyase 1 (*Bxpel1*) from *B. xylophilus* was amplified by PCR using cDNA templates. The amplified fragments were cloned into the Pmd™18-T Vector and then amplified by PCR using T7-labeled gene-specific primers (Table 2), before generating a double-stranded template where the ends were defined by the T7 promoters themselves. *In vitro* transcription was performed to produce dsRNA using a MEGA script RNAi kit (Ambion, Austin, TX, USA), according to the manufacturer’s instructions. About 8000 juveniles at the second stage of *B. xylophilus* were soaked in 200 μL M9 buffer (16.9 mM Na_2_HPO_4_, 8.8 mM KH_2_PO_4_, 34.2 mM NaCl, and 0.4 mM MgSO_4_, pH 7.0) containing 1 mg/mL dsRNA and incubated in a rotator (200 rpm/min) in the dark at 20 °C for 48 h. A mixture of *B. xylophilus* soaked in the same solution without dsRNA was used as the control. After soaking, the nematodes were centrifuged at 3000 rpm for 5 min and then washed three times with sterile water. There were three replicates for each treatment. The morphology of nematodes was observed using a compound microscope (Olympus CX31, Tokyo, Japan).

**Table 2:** PCR primers for the *Bxpel1* gene in *B. xylophilus*. The underlined primers are the sequences of T7 promotor.

Gene Name	Forward Sequence 5′–3′	Reverse Sequence 5′–3′
*Bxpel1*-T7	TAATACGACTCACTATAGGG	TAATACGACTCACTATAGGG
	TTGTTTCGGCTCAGTTTGGA	TTTGGGTTCCTGGTTGTTGT

### 4.4. The Expression of Bxpel1 Gene after B. xylophilus Soaking in dsRNA

Real-time quantitative RT-PCR (qRT-PCR) was used to assess the mRNA expression levels of the *Bxpel1* gene in *B. xylophilus*. Gene-specific primers were designed using Primer Premier 5 software (Premier Biosoft Int., Palo Alto, CA, USA) (Table 3). The actin gene in *B. xylophilus* was selected as the internal control [27]. Twenty thousand nematodes were used to extract total mRNA and the concentration of cDNA used as template was 40 ng/μL. RT-PCR was conducted using a 20 μL reaction volume, which contained 2 μL of template, 10 μL SYBR Premix Ex Tap, 0.4 μL ROX Reference Dye II, 0.4 μL forward primer, 0.4 μL reverse primer, and 6.8 μL ddH_2_O. The cycling conditions comprised one cycle of denaturing at 94 °C for 30 s, followed by 40 cycles of denaturing at 94 °C for 5 s and then annealing and extension at 60 °C for 34 s. The expression of the *Bxpel1* gene was detected by Real-time PCR analyzer (ABI 7500, Darmstadt, Germany).

**Table 3:** Primers used for quantitative real-time PCR (qRT-PCR) analysis.

Probe Name	Forward Sequence 5′–3′	Reverse Sequence 5′–3′
Actin	GCAACACGGAGTTCGTTGTA	GTATCGTCACCAACTGGGAT
*Bxpel1*	ACCATCAAGAATTTCCAGGTAG	TTTCCGCAAGAACGATAGAG

### 4.5. Effect of RNAi on Reproduction of B. xylophilus

In our study, 10 μL (200 nematodes) suspension of the second stage of *B. xylophilus* were soaked in the same volume of control solution, *Bxpel1* dsRNA solution, ddH_2_O and *GFP* gene dsRNA for 48 h, respectively. The nematodes were washed three times with sterile water and transferred onto a PDA plate with *B. cinerea*. After cultivating at 25 °C for 5 days, the nematodes were isolated by Baermann funnels and collected by centrifugation at 3000 rpm for 5 min and counted by compound microscope (Olympus CX31, Tokyo, Japan).

### 4.6. Effect of RNAi on the Pathogenicity of B. xylophilus

After soaking, the nematodes were inoculated into *P. thunbergii* where three treatments were tested: (i) 200 μL suspension of juvenile nematodes soaked in the control solution was piped into wounds (2 cm in length) on *P. thunbergii* seedlings at about 30–50 cm above the soil level; (ii) the same volume of control solution without nematodes was piped into wounds on seedlings; or (iii) the same volume of nematodes soaked in *Bxpel1* dsRNA solution was inoculated into seedlings. Wilting symptoms development of *P. thunbergii* after inoculation with *B. xylophilus* was observed at 1, 10, 20 and 30 days. The number of dead seedlings was counted according to the serious wilting symptom, with all the needles becoming yellow. The mortality rates were calculated as follows:

$$\text{Mortality rates}=\frac{\sum \text{number of dead seedlings}}{\text{Total number of seedlings}}\times 100\%$$

### 4.7. Effect of RNAi on the Dispersal of B. xylophilus

A 200 μL volume suspension of *B. xylophilus* (the second stage) soaked in *Bxpel1* dsRNA solution was inoculated into *P. thunbergii*, and 200 μL of *B. xylophilus* soaked in sterile water was inoculated into *P. thunbergii* as the control. Three seedlings were cut for each treatment every 4 days. The branches and leaves were removed from the seedlings and the trunks were cut 5 cm above and below the site of inoculation. *B. xylophilus* was then collected from the cut trunk using Baermann funnels and the number of nematodes was counted.

### 4.8. Statistical Analysis

Analyses were performed using the SPSS statistical software package (Version 16.0) (International Business Machines Corp., Chicago, IL, US). All data are presented as the mean value ± standard deviation (SD) of three replicates. The significances of experimental effects of dsRNAi on reproduction and expression of *B. xylophilus* on *B. cinerea* were tested by a one-way analysis of variance (Tukey’s test). The significances in Table 1 about the migration and reproduction of *B. xylophilus* in pine trees were analyzed by students’s *t*-test.

## 5. Conclusions

In summary, the results of our study demonstrated that *B. xylophilus* is susceptible to RNAi, and that *Bxpel1* plays a critical role in the PWD process. Our results suggest that silencing of the *Bxpel1* gene had a negative effect in the reproduction of *B. xylophilus* and ultimately in their migration within the host tree. In addition, *Bxpel1* RNAi decreased the pathogenicity of *B. xylophilus.* However, the effect of *Bxpel1* RNAi vanished over time in the PWNs. The successful silencing of *Bxpel1* in this study suggests the possibility of more widespread applications of RNAi in PWNs as well as providing insights into the molecular mechanism of PWD. The present study may provide a reference for the functional analysis of genes in *B. xylophilus* and facilitate the development of effective control strategies to combat PWNs.
